# Evaluation of the neuroprotective potential of optimized intranasal polydopamine nanoparticles in a lipopolysaccharide-induced rat model for Alzheimer’s disease management

**DOI:** 10.1038/s41598-025-22844-z

**Published:** 2025-11-04

**Authors:** Reem R. Ibrahim, Abeer Salama, Samar M. Abouelatta, Mona Elhabak

**Affiliations:** 1 Department of Pharmaceutics and Industrial Pharmacy, Faculty of Pharmacy, Helwan University, Helwan, Cairo Egypt; 2 Pharmacology Department, National Research Centre, El- Buhouth St., Dokki, Cairo 12622 Egypt; 3 Department of Pharmaceutics and Industrial Pharmacy, Faculty of Pharmacy, Ahram Canadian University, 6 October, Cairo Egypt

**Keywords:** Polydopamine nanoparticles, Sodium taurocholate, Alzheimer’s disease, Nasal delivery, Brain targeting, Health care, Medical research, Nanoscience and technology

## Abstract

Alzheimer’s Disease (AD) and related dementias are on the rise globally, with forecasts suggesting that the case number will triple in the next three decades, particularly in Africa and the Middle East. This study aimed to prepare brain-targeted highly permeable intranasal polydopamine nanoparticles (PDA NPs) and to investigate their neuroprotective effect in AD. PDA NPs were fabricated via dopamine hydrochloride (DA HCl) polymerization using various safe and nontoxic surfactants to reduce particle size (PS) and enhance nasal penetration. A 4^1^*2^2^ full factorial design was employed. The optimized formulation was composed of 10 mg DA HCl and sodium taurocholate as a surfactant at pH 10. The optimized formulation was subjected to in vitro characterization: PS, polydispersity index (PDI), zeta potential (ZP), Transmission Electron Microscopy (TEM), Fourier transform infrared spectroscopy (FTIR), in vitro cell cytotoxicity, and in vivo evaluation: in vivo tissue uptake using confocal laser scanning microscopy (CLSM) and investigation of neuroprotective effect in lipopolysaccharide-induced rat model. The optimized formulation produced minimal PS (111.3 nm), PDI (0.324), and maximum ZP (− 36.87 mV). PDA NPs showed high brain uptake and significant behavioral and biochemical results on rats as well as an elevation of dopamine expression and ameliorated neuron degeneration. PDA NPs exerted a neuroprotective effect in Alzheimer’s disease through their anti-inflammatory and ACh/dopamine stimulation effects.

**Supplementary Information:**

The online version contains supplementary material available at 10.1038/s41598-025-22844-z.

## Introduction

 People suffering from central nervous system diseases are vastly increasing, affecting human survival, and increasing health care costs. Neurological disorders are the main reason for global disability-adjusted life-years (DALYs). Central nervous system (CNS) diseases affect the structure and function of the neurons and the brain, usually classified as acute brain injury and chronic neurodegenerative diseases (NDs)^1^.

The most frequent age-linked neurodegenerative disorder, depicted by advanced deterioration of cognitive ability is Alzheimer’s disease (AD). In correspondence to the International Alzheimer’s Association (2018) 50 million people or more suffer from AD globally^2^. The main characteristic of AD is the manifestation of extracellular amyloid β (Aβ) plaques and intracellular neurofibrillary (tau) tangles^3^. Usually, AD begins with short-temporary memory loss, followed by cognitive loss, behavioral chaos, difficulties in coordination subsequent premature death occurs^4^. The main challenge in dealing with AD is that most of the systemically administered drugs can’t penetrate in the brain owing to the inability to penetrate through the blood-brain barrier (BBB)^5^.

The most confronting barrier for the drugs to reach the desired tissue is the BBB. The use of nanomedicines has shown important returns in its application for instance their capability to encapsulate large number of drug molecules, extend drug release rate, enhanced targeting to a specific tissue via surface modification, thus allowing even the transport across BBB, and enhance drug bioavailability with lowering its toxicity^6^.

These systems are more competent in delivering drugs to brain via the trigeminal and olfactory nerves thus avoiding BBB. Furthermore, drug delivery is frequently made by extended infusion or highly invasive methods. Because of all these obstacles, the obtained results for the treatment of these disorders are poor. The approach to bypassing the BBB by using the intranasal delivery of drugs is now vastly adopted^6^. These delivery systems are very efficient in delivering drugs noninvasively to the brain *via* the olfactory and trigeminal nerves from the nasal olfactory epithelium thus avoiding BBB. Besides, it is simply used, avoids first-pass metabolism, is cost-effective, and affords immediate therapeutic drug levels in the brain^5^.

Upon correlating dopamine receptors (control of mood and emotional stability) with AD it was observed that hippocampal D2R is responsible for memory functioning. Dopamine exploits *via* the (D2-corresponding receptors) enhance cortical impulsiveness and (D1-corresponding receptors) tend to improve cortical acetylcholine release^7^. From the previous findings, it was suggested that if low levels of dopamine were delivered to the hippocampus new memories won’t be recognized indicating a high risk of dementia and Alzheimer’s.

Dopamine (DA HCl) is a prominent catecholamine neurotransmitter that performs a substantial role in the human different systems (central nervous, renal, hormonal, and cardiovascular). The polymerization of DA HCl produced Polydopamine (PDA) which displays exclusive chemical features that have lately captivated scientists in the biomedical field. PDA chemical structure which is comparable to the structure of melanin nominates PDA as a promising biocompatible and biodegradable polymer^8^. The loading of dopamine in different nanosystems for intranasal administration for management of neurodegenerative diseases was previously reported^9–11^. In recent research, the preparation of PDA NPs represents a challenge to control the self-assembly process and hence the morphology and PS. PDA NPs were employed as neurotoxic metal chelates and inhibitors for the regulation of Aβ aggregation thus alleviating AD pathological conditions, this occurs *via* both covalent and noncovalent interactions^12^.

This study aimed to optimize dopamine polymerization by employing different surfactants and pH to achieve suitable particle size and enhanced permeation for brain targeting. The innovation in this research stems from the investigation of previously unused surfactants in PDA preparation. Our study focused on using good tolerability, low toxicity, and non-irritating surfactants for the nasal mucosa. Our goal is to achieve brain targeting of PDA NPs via the intranasal route. The novelty of this research also lies in the in-vivo evaluation of neuroprotective effect of PDA NPs in rat model compared to free dopamine.

## Materials and methods

### Materials

Dopamine hydrochloride 99% (DA HCl) was supplied by Acros Organics (GmbH, Darmstadt, Germany). Sodium Taurocholate was purchased from MP Biomedicals, LLC (Illkirch, France). D-α-Tocopherol polyethylene glycol 1000 succinate (TPGS) was given by Isochem (France) as a gift. Pluronic PL-F407 was obtained as a gift from BASF SE (Ludwigshafen, Germany). Lipopolysaccharide (LPS) was purchased from Sigma Aldrich Chemical Co., USA. Urea and creatinine were purschased from Biodiagnostic Co., Egypt. Nuclear factor- κB (NF-κB), Tumor necrosis factor-α (TNFα), acetylcholine (ACh), and dopamine kits were obtained from Sunlong Biotech Co., Ltd, China. The used buffer (Tris- HCl) (2-Amino-2-hydroxymethyl-propane-1,3-diol) Semi-permeable membrane tubing (Spectra Por©; MWCO 12,000–14,000) was procured from Spectrum Laboratories Inc., (Rancho Dominguez, CA) and all used chemical reagents were of analytical grades.

### Methods

All methods were carried out in accordance with relevant guidelines and regulations.

### Preparation of bio-inspired self-assembled polydopamine nanoparticles (PDA NPs) utilizing mixed full factorial design

Self-assembled PDA NPs were simply formulated by oxidation of DA HCl solution as per Wang et al.^13^. A definite amount of DA HCl was dissolved in Tris- HCl buffer solution containing 0.5% (w/v) surfactant and the pH of the solution was adjusted to predetermined pHs. The mixture was stirred at room temperature overnight at 100 rpm to allow the polymerization of DA HCl.

Different PDA NPs formulations were prepared according to 4^1^*2^2^ full factorial design (Design Expert 7^®^, https://design-expert2.software.informer.com/7.0/). The designated independent variables were the amount of DA HCl (A), pH (B), and kind of surfactant (C). The amount of DA HCl and the pH were varied at two levels (5 and 10 mg) and (7 and 10) respectively. The type of surfactant was studied at four levels (none, TPGS, sodium taurocholate, and PL-F407). The selected considered responses were the particle size (PS) (Y_1_) along with Zeta potential (ZP) (Y_2_) as observed in Table 1. The different formulation parameters are illustrated in Table 2.

**Table 1:** Mixed factorial design 4^1^*2^2^ used for the optimization of synthesis of bioinspired polydopamine systems.

Factors (independent variables)	Levels			
A: Amount of DA HCl (mg)	5 mg		10 mg	
B: pH	7		10	
C: Type of Surfactant	None	TPGS	Taurocholate	Pluronic F-407
Responses (dependent variables)	Constraints			
Y1: PS	minimize			
Y2: ZP	maximize			

DA HCl; Dopamine HCl, PS, particle size; ZP, zeta potential.

**Table 2:** Conditions proposed for synthesis of the bioinspired polydopamine systems and their observed studied responses.

#	Amount of DA HCl (mg)	pH	Type of surfactant	PS	PDI	ZP
F-1	5	7	NONE	749.25 ± 2.90	0.69 ± 0.09	-14.34 ± 1.19
F-2	5	7	TPGS	151.3 ± 4.67	0.51 ± 0.02	-11.03 ± 1.16
F-3	5	7	Taurocholate	899.05 ± 102.6	0.75 ± 0.06	-11.2 ± 0.99
F-4	5	7	Pluronic F-407	103.75 ± 11.53	0.27 ± 0.02	-5.84 ± 0.35
F-5	5	10	NONE	533.65 ± 14.92	0.48 ± 0.01	-22.15 ± 0.78
F-6	5	10	TPGS	304.75 ± 11.38	0.69 ± 0.08	-25.7 ± 0.14
F-7	5	10	Taurocholate	581.05 ± 18.46	0.60 ± 0.06	-35.1 ± 3.82
F-8	5	10	Pluronic F-407	268.9 ± 12.16	0.67 ± 0.07	-8.99 ± 0.95
F-9	10	7	NONE	803.2 ± 10.47	0.23 ± 0.02	-9.38 ± 1.64
F-10	10	7	TPGS	99.04 ± 5.61	0.25 ± 0.01	-3.82 ± 0.86
F-11	10	7	Taurocholate	1335 ± 47.09	0.30 ± 0.02	-4.64 ± 0.96
F-12	10	7	Pluronic F-407	82.65 ± 6.07	0.34 ± 0.02	-2.25 ± 0.83
F-13	10	10	NONE	744.9 ± 15.56	0.44 ± 0.01	-20.6 ± 1.13
F-14	10	10	TPGS	23.94 ± 1.29	0.25 ± 0.03	-25.6 ± 0.99
F-15	10	10	Taurocholate	98.11 ± 7.76	0.38 ± 0.08	-38.25 ± 1.63
F-16	10	10	Pluronic F-407	89.16 ± 2.07	0.70 ± 0.01	-9.94 ± 0.11

DA HCl; Dopamine HCl, PS, particle size; PDI, polydispersity index; ZP, zeta potential.

*Mean ± Standard deviation (*n* = 3).

**Table 3:** The observed and predicted values of the optimized bioinspired polydopamine systems.

Factor	Optimized level (D = 0.912)		
A: Amount of DA HCl (mg)	10 mg		
B: pH	10		
C: Type of Surfactant	Taurocholate		
Response	Expected	Observed	Residual*
Y_1_:PS	98.11	111.3	-13.19
PDI	0.383	0.324	0.059
Y_2_: ZP	-38.25	-36.87	1.38

*Residual = expected value – observed value.

### In vitro polydopamine nanoparticles characterization

#### Determination of PS, PDI, and ZP

The mean PS, and PDI of the prepared PDA NPs were measured using Zetasizer Nano ZS (Malvern Instruments, UK) employing dynamic light scattering technique (DLS). The ZP of the samples was analyzed using doppler electrophoresis laser velocimetry using Zetasizer (Nano ZS, Malvern, UK). Samples were adequately diluted with purified water and vortexed for 30 s before measurements. All results were shown as average ± standard deviation (SD)^14^.

#### Study of the used mixed factorial design and optimization of all formulation variables

The PS and ZP findings were investigated using Design Expert^®^7 software. Optimal formulation conditions were reached and the optimal formulation with minimized PS and maximized ZP was prepared and characterized. The optimal formulation was proposed according to the desirability (the nearest to 1) which had a minimal PS and maximal ZP (absolute value).

### Characterization of the optimal PDA NPs formulation

#### Transmission electron microscopy (TEM)

The structure and the size of the optimal PDA NPs formulation was observed via Joel TEM (JEM 1230 TEM, Tokyo). An amount of PDA NPs dispersion was carefully placed on the TEM metallic grid, left for drying at 25 ± 2 °C, and then imaged by TEM^15^.

#### Fourier transform infrared spectroscopy (FTIR)

The FTIR spectra of the samples were scanned using an IR Spirit spectrophotometer (Shimadzu, Japan) to study any possible chemical interactions between DA HCl, and the used ingredients and to confirm the formation of PDA NPs. The spectra of each DA HCl, sodium taurocholate, their physical mixture, and the optimized PDA NPs formulation were measured through the range of 4000–400 cm ^−1^^16^.

### In vitro cell cytotoxicity

To evaluate the safety of the optimal formulation in vitro cell cytotoxicity was performed. MTT assay was applied using an oral epithelial cell line (Nawah-Scientific, Egypt). These cells were incubated in 96 well plates in Dulbecco’s Modified Eagle Medium (DMEM) complemented with 100 units/mL of penicillin, 100 mg/mL of streptomycin, and 10% of fetal bovine serum, at 37 °C in 5% CO_2_ (v/v) humidified atmosphere for 24 h. Aliquots of 100ul cell suspension were added to 96 well plates then incubated at 37 °C in complete media for 24 h in a 5% CO_2_. The optimal PDA NPs were diluted in media to make 10 different concentrations (1–10000 µg/mL). A volume of 100ul of media containing different PDA concentrations was added to the cells. After 24 h incubation, the media was discarded and replaced with 20 µL MTT (1 mg/ml) mixed with 100ul phosphate buffer saline (PBS) in each well and kept at 37 °C for 4 h. Then, 100ul DMSO was added, and the absorbance at a lambda max of 570 nm was measured using a microplate reader (BMG LABTECH FLUOstar^®^ Omega, Germany). From the dose-response graph, IC50 was determined *via* Graph Pad Prism^®^ software(https://graphpad-prism.software.informer.com)^17^.

### In vivo studies

#### Animal subjects

Male albino Wistar rats, weighing 120–140 g, were obtained from the National Research Centre (NRC; Cairo, Egypt). During the experimental period, the rats were maintained in a quiet place with free food and water access at a temperature of 20 ± 1 °C on a 12/12-h light/dark cycle. The experimental protocol was approved with an Ethical Approval No. (REC 0623) by Ethics, and Research Committee, Faculty of pharmacy, Ahram Canadian University. All used procedures tracked the ARRIVE guidelines recommendations.

#### Evaluation of PDA NPs in vivo cell uptake with the laser confocal scanning microscopy (LSM)

Rhodamine-labeled PDA NPs were used to study NPs uptake in the brain using laser confocal scanning microscopy **(**Model: LSM 710, Software Version: ZEN 2.3, Carl Zeiss, Jena, Germany). The rhodamine-labeled PDA NPs were formulated using the same method of preparation described formerly with slight modification^13^. DA HCl (10 mg) was dissolved in 10 ml Tris- HCl buffer solution containing 0.5% (w/v) sodium taurocholate and rhodamine dye (1 mg/ mL) under mixing at 100 rpm overnight. To ensure removal of free rhodamine, rhodamine labelled PDA NPs were dialyzed against purified water using dialysis semi-permeable membrane with several water changes for 8 h. Six Wistar male rats (120–140 g) were randomly divided into two separate groups (3 rats per group). A volume of 50 µL rhodamine dye solution at a concentration of 1 mg/mL and the PDA dye loaded nanoparticles were applied *via* the intranasal route to the first and second groups, respectively. Two hours after treatment, the rats were decapitated, and the brain was removed, swept using normal saline, cut then fixed into slices, and then examined using CLSM with an Ar excitation laser at a lambda max of 543 nm. Images were handled and analyzed via ZEN 2.3 software^18^.

#### Experimental design

All experimental groups consisted of an equal number of male albino Wistar rats, divided into 4 groups (10 per group) to ensure consistency and statistical reliability. Initially, group 1: Negative control rats received normal saline. Then, group 2: Positive control group induced by LPS (250 µg/kg) one time/day for 1 week; intraperitoneal (IP)^17^. Moreover, group 3: rats received LPS (250 µg/kg) and 100 ul of DA HCl solution (10 mg/ml) simultaneously intranasally once daily for 7 days. Finally, group 4: rats administered LPS (250 µg/kg) and 100ul of PDA NPs (10 mg/ml) simultaneously intranasally once a day for 7 days.

#### Evaluation of behavioral activity using y-maze

The Y-maze apparatus consisted of three identical arms identified as A, B, and C (each 40 cm long, 10 cm wide, and 18 cm high) arranged at an angle of 120° from each other. A training stage was carried out in 8 min. After 24 h, the rats were allowed to convey for 8 min in the second stage and their behaviors were recorded blindly. The change number represents the successive entrances into 3 varying aisles in 3 overlying sets (for example ABCBACA…)^18^.

#### Biochemical evaluation

By the end of the experimental period, rats were sacrificed by decapitation and their brains were excised and retained in freezing phosphate buffer (pH 7.4) for preparation of 20% homogenate, employing a tissue homogenizer (MPW_120, Bit-Lab Medical instruments, Poland). Afterwards, these tissues were subjected to centrifugation at 4000 rpm/min for 10 min at 4 °C using cooling centrifuge (Laboratory Centrifuge, 2 K15, Sigma Co., Germany). The supernatant was collected and kept at − 80 °C^19^ for further quantification of NF-κB, TNFα, Ach, and dopamine using ELISA kits^20^.

#### Statistical analysis

Statistical analysis SPSS statistical software package (SPSS 13.0® for Windows; SPSS, Inc., USA, https://www.uhi.ac.uk/en/lis/software-downloads/spss-statistics-home-use/) was used for performing statistical analysis. The data were expressed as mean ± SD. Statistical significance was evaluated by one way analysis of variance, followed by Fisher’s LSD at P less than 0.05.

#### Histopathological study

Brain tissues were randomly taken from each group and fixed in neutral buffered formalin (10% w/v), then washed with water and dehydrated with serial dilution of alcohol. Finally, they were embedded in paraffin bees wax blocks. Tissues sections of 5 μm thickness were fixed on glass slides and stained with hematoxylin and eosin (H&E).

## Results and discussion

The PDA NPs were prepared by adopting self-polymerization technique using DA HCl as starting material in tris buffer. The effect of the amount of DA HCl, pH, and the type of surfactant on PS and ZP were studied.

### Characterization of self-assembled polydopamine nanoparticles

While analyzing the general full factorial design, the adequate precision ratio values above 4 and for all responses, the difference between the predicted R^2^ values and the adjusted R^2^ values, was detected to be lower than 0.2.

#### Effect of the independent variables on PS

**Fig. 1:**
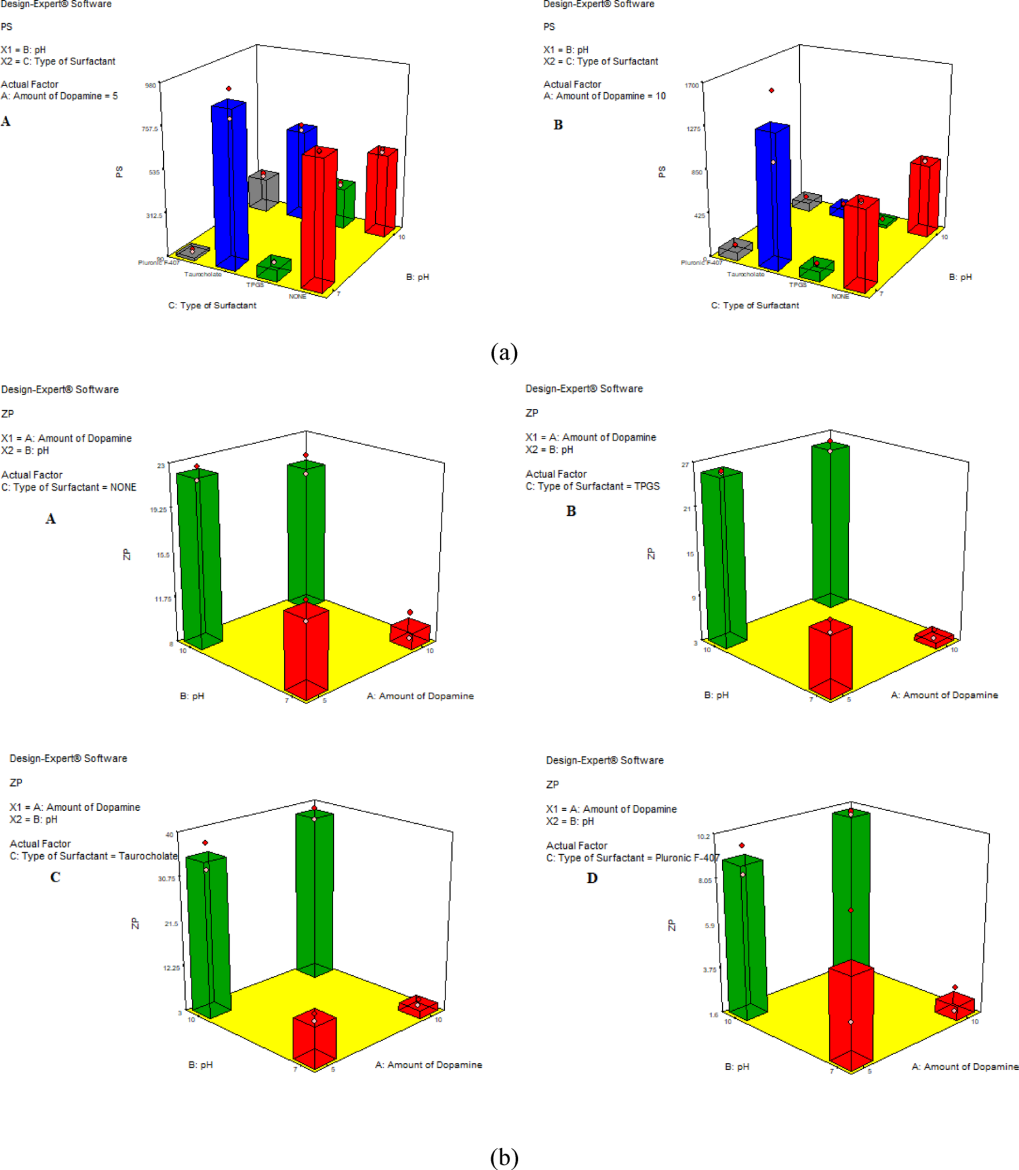

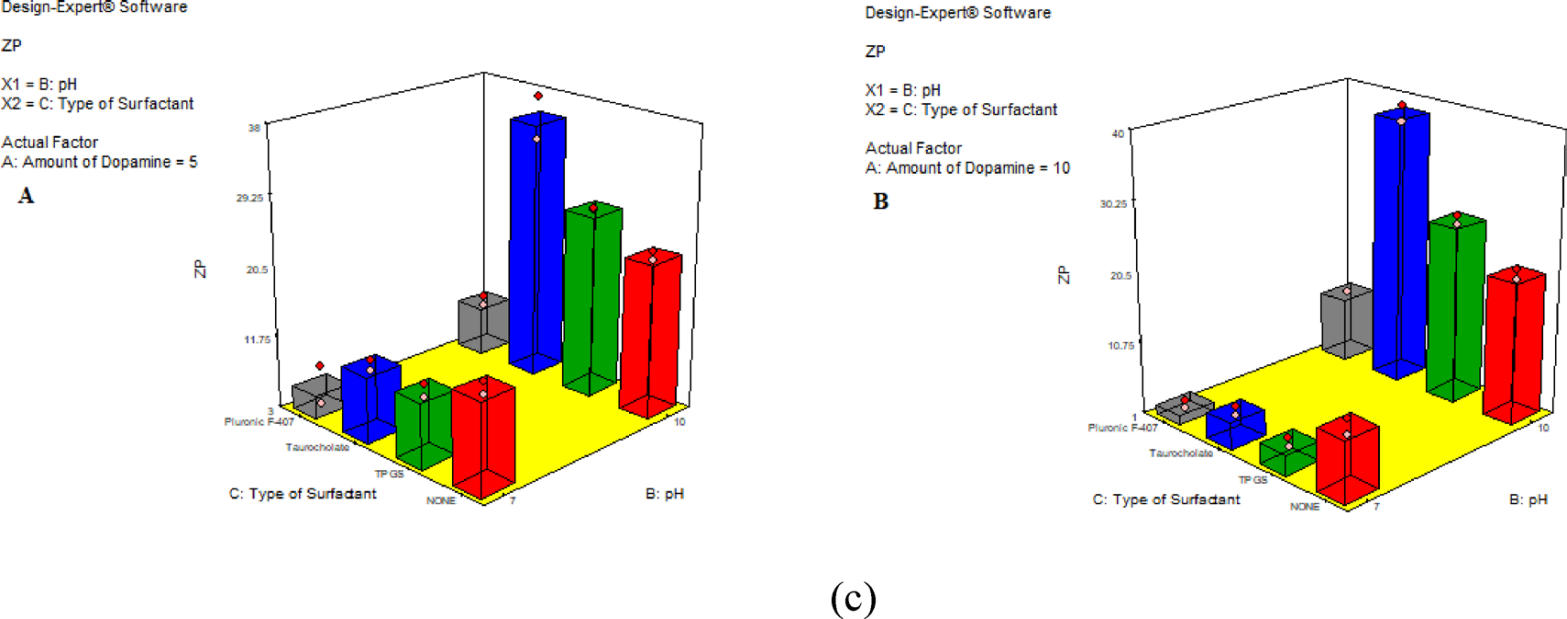
(**a**) Response surface plot elucidating the effect of the studied factors on PS. (**b**) Response surface plot elucidating the amount of DA HCl added on ZP. (**c**) Response surface plot elucidating the type of surfactant on ZP.

The Pareto charts displayed in (Fig. 1a) revealed that the amount of DA HCl (A) was insignificant (*p* > 0.05) whereas the pH (B) showed an antagonistic significant effect. The type of surfactant (C) was significant (*p* < 0.05). The mean PS of PDA NPs was found to be in the range 23.94 ± 1.29 to 1335 ± 47.09 nm as displayed in Table 2. Upon further statistical analysis, one-way ANOVA revealed that both pH and type of surfactant significantly affected the PS (*p* < 0.05), whereas the amount of dopamine was insignificant. Increasing pH from 7 to 10 was accompanied by a significant decrease in PS for the formulations prepared without surfactants. Similar results were reported by *Ball*; PDA NPs prepared using DA HCl revealed a pH-dependent size. The PS decreased with increasing the pH to 10 to reach tens of nanometers^21^. The large PS obtained at pH 7 might be due to the formation of hydrogen bonds between catechol groups. The deprotonation of the functional groups obtained with increasing pH to 10 possibly led to a decrease in PS^22^.

The addition of surfactants significantly decreased PS (*p* < 0.05) except taurocholate; when compared to the formulation prepared without surfactant at the same pH and with the same amount of dopamine. The addition of taurocholate displayed a significant rise (*p* < 0.05) in particle size except when prepared at pH 10, using 10 mg dopamine, PS decreased from 744.9 ± 15.56 to 98.11 ± 7.76 nm (Table 2). Previous studies had shown the ability to control the self-assembly of PDA by adding polymers, proteins, and surfactants with long aliphatic chains in the dopamine solution. Polyvinyl alcohol rich in hydroxyl groups provides steric stabilization when adsorbed on the surface of PDA NPs. Surfactants due to their amphiphilic properties could interact with the 5,6-dihydroxy indole based oligomers formed via the process of dopamine oxidation and hence affect their self-assembly^23^. It was also previously reported that anionic and cationic surfactants accelerated dopamine oxidation and permitted a reduction in PDA aggregates size. The hydrodynamic diameter of PDA NPs is somewhat larger than that of the micelles formed exceeding the critical micellar concentration of the used surfactant. This might explain the difference in PS obtained with varying types of surfactants used. Contrary to our results, it was stated that nonionic and anionic surfactants having short aliphatic chains were not able to control the self-assembly of PDA^21^. The decrease in PS of PDA NPs could be attributed to various factors beyond the type of surfactants, including critical micelle concentration and HLB.

The PDI values were varying between 0.27 ± 0.02 and 0.70 ± 0.01 as shown in Table 2. Results showed a big discrepancy in PDI depending on the composition and the conditions of preparation.

#### Effect of the independent variables on ZP

**Fig. 2:**
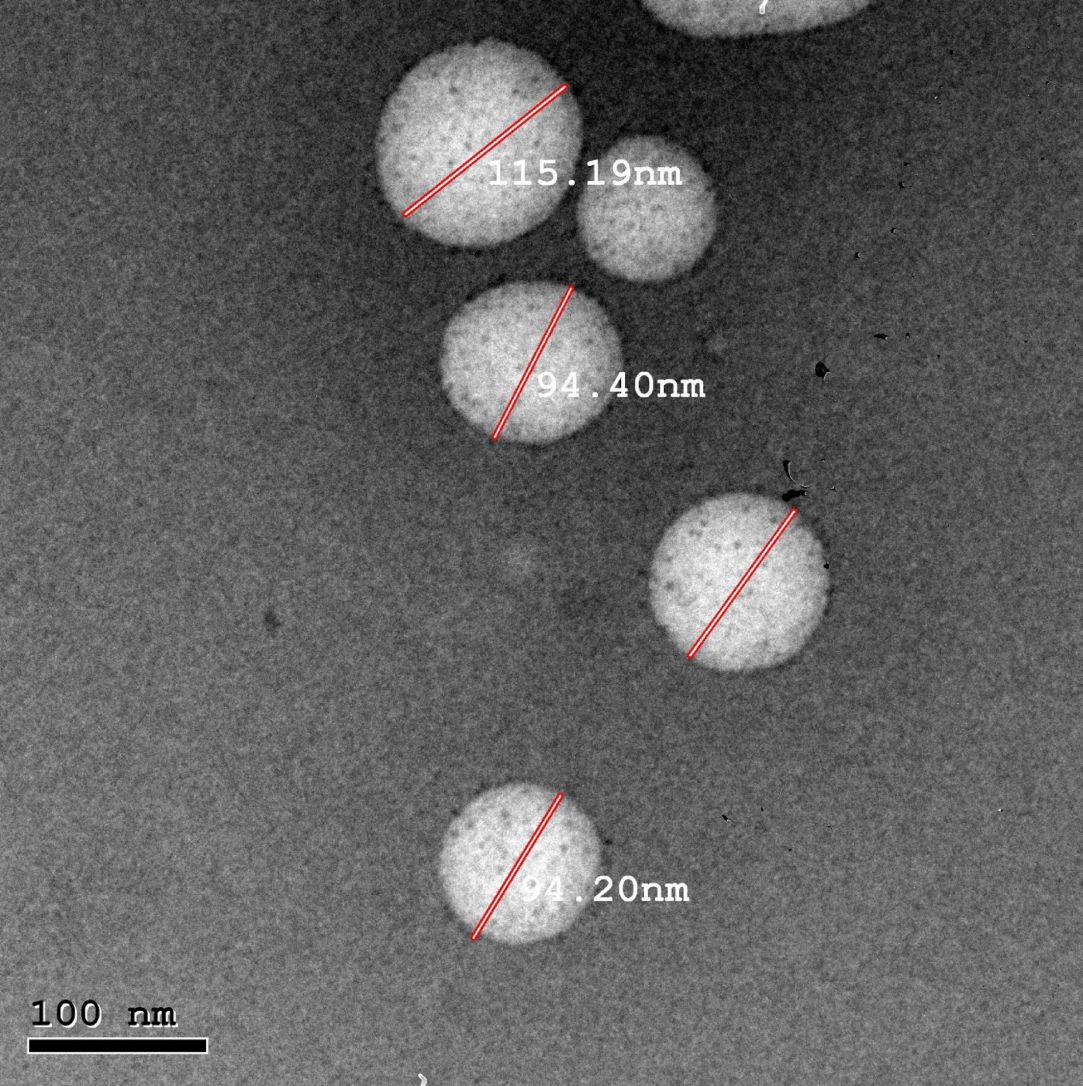
TEM micrographs of the optimized PDA NPs (F-15) synthesized by self-polymerization in tris buffer at pH = 10.

The stability of the PDA was estimated by measuring the ZP of each preparation. The prepared formulae showed ZP ranging from − 2.25 ± 0.83 to -38.25 ± 1.63 mV as depicted in Table 2. The 3D surface plot displayed in (Fig. 1b and c) showed that the studied factors, namely, the amount of DA HCl (A), pH (B), along with the type of surfactant (C) were all statistically significant (*p* < 0.05). As shown in Fig. 2, the ZP was antagonistically affected by the increasing amount of dopamine at pH = 7. Whereas pH = 10, the amount of dopamine was insignificant (*p* > 0.05) concerning all the studied types of surfactants on ZP. Different mechanisms of PDA interaction with aqueous solutions were stated in the literature. Hence, PDA binds in different ways, different chemical groups are exposed. The amine group is exposed if PDA binds with the quinone OH− groups and the negativity of the surface charge decreases^24^. This might explain the decrease in ZP with increasing the amount of dopamine in the solution. Contrary to our results, Sherwood et al. reported higher negative ZP with increasing DA HCl amount as it could impart more negative group to the surface^22^.

From Table 2, it was also obvious that the increase in pH from 7 to 10 was accompanied by an increase in ZP. The positive and negative charge of the PDA resulted from the protonation/deprotonation of both the catechol and amine groups found on its surface. As the isoelectric point of PDA is around 4, PDA acquires an overall negative charge at higher pH^25^. It was reported in the literature that ZP values were around − 4.58 and − 39 mV when prepared in Tris buffer at pH 7 and 8.5 respectively^26^. Increasing pH is accompanied by an increase in the negative charge due to the catechol group deprotonation that could happen at higher pH^22^.

The effect of surfactant could be arranged according to the charge it imparts to the surface of PDA as follows: taurocholate > TPGS > Pluronic F-407. Taurocholate, owing to its anionic nature, imparts a larger negative potential to the NPs surface^27^. At pH 7, surfactants significantly lowered the absolute value of ZP; while at pH 10, taurocholate and TPGs significantly increased the negativity of ZP. This difference in the effect of surfactant on ZP obtained with changing pH from 7 to 10 might be due to the surfactant effect on PDA binding mechanism as well as the exposed groups.

### Optimization of the design

A desirability function equal to 0.912 was employed for the design optimization. The optimal formulation (F15) was prepared with 10 mg DA HCl and 0.5% (w/v) sodium taurocholate at pH = 10. Table 3 assures the design validity by computing the residual. Ten milligrams of DA HCl were dispersed in Tris-HCl buffer of pH = 10 containing 0.5%(w/v) taurocholate as a surfactant. The suggested optimized formulation exhibited small PS (111.3 nm) with narrow PDI (0.324) and ZP of -36.87 mV indicating the stability of the formed PDA NPs that will go further analysis as a promising brain targeting drug delivery systems.

### Characterization of the optimal formulation

#### Transmission electron microscopy (TEM)

TEM photo of the optimal formulation (F15) is presented in (Fig. 2). Particles appeared spherical with a smooth surface. The average PS obtained agreed with that measured using dynamic light scattering with zeta sizer apparatus.

#### Fourier transform infrared spectroscopy (FTIR)

FTIR analysis was conducted on DA HCl, taurocholate, and synthesized PDA NPs to identify the functional groups on PDA to confirm succeeded synthesis. (Figure S1, supplementary data) displays the IR spectra of DA HCl, taurocholate, and PDA NPs. Dopamine HCl showed distinct peaks at 3373, 3253.73, 3042.58, 1615, and 1495 cm^−1^ corresponding to the N-H stretching of amines, intermolecular hydrogen bonding of the catechol group, aromatic C–H stretching, N–H bending vibrations and aromatic ring stretching respectively^28^. PDA NPs spectrum revealed a wide band at 3423 cm^−1^ confirming the intermolecular hydrogen bonds between oligomers that composes the structure of PDA^28^. The peaks of polyindole aromatic ring stretching vibrations appeared at 1615 cm^−1^ and 1495 cm^−1^. The FTIR spectrum of PDA NPs showing the presence of indole structure confirmed the successful DA HCl polymerization to PDA^29^. The sodium taurocholate spectrum showed bands of CH and COO− stretching vibrations at 2939.78, 2888.4, and 1552.66 cm^−1^ respectively. The physical mixture revealed the characteristic peaks of dopamine and taurocholate.

### In vitro cytotoxicity

**Fig. 3:**
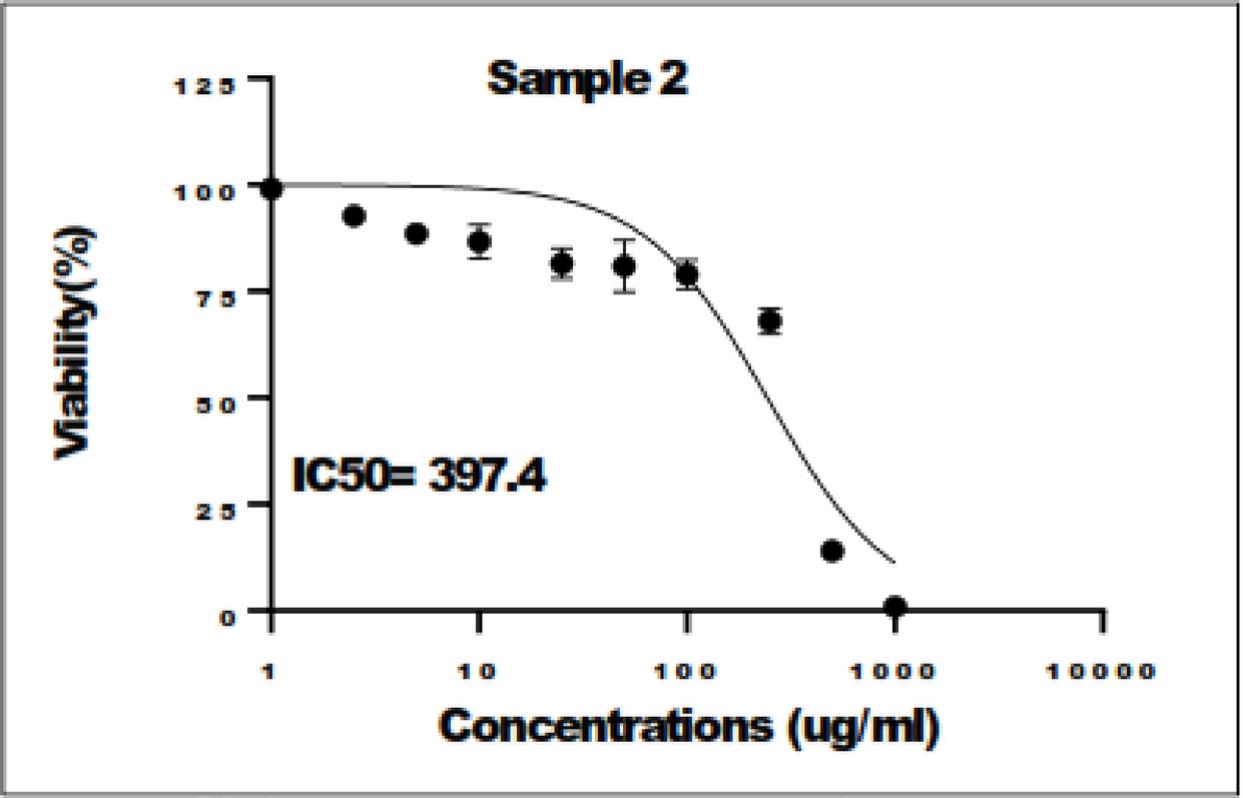
Cytotoxicity results of the optimized formulation (F15) against oral epithelial cell line (OEC) using MTT assay.

To assess the optimal formulation’s safety on the oral epithelial cell line MTT assay was employed. The cell viability percentage is illustrated in (Fig. 3). Results showed dose-dependent cytotoxicity for all the tested samples. The optimized PDA NPs exhibited a poor cytotoxic effect on oral epithelial cell lines with IC50 equal to 397.4 µg/mL. Nordin et al. observed the effect of PDA NPs on the viability of stroma as well as human breast, colon, and liver carcinoma cells. Results revealed that the viability of stromal cells was higher than 80% when treated for 72 h with 0.042 mg/mL PDA NPs (100 nm)^30^. On the contrary, the viability of human breast, liver, and colon carcinoma cells was reduced to 59, 60, and 63% respectively. PDA NPs’ effect on the viability of stromal cells is different than on cancer cells. The Iron affinity of PDA NPs and ferroptosis could be responsible for the cytotoxicity of PDA NPs in tumor cells^31^. The low cytotoxicity of PDA NPs is a valuable indicator of their biocompatibility with oral epithelial cells and hence, PDA NPs could be considered an intriguing drug delivery candidate.

### In vivo cell uptake using laser confocal scanning microscopy (LCSM)

**Fig. 4:**
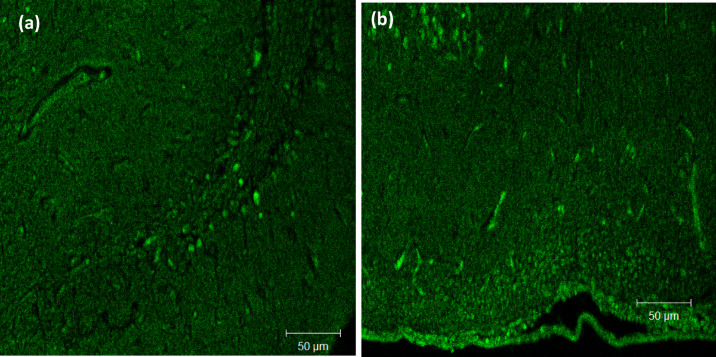
Confocal images of brain tissue after I.N. administration of (**a**) Rhodamine B labeled PDA NPs and (**b**) Rhodamine solution.

The LCSM is an imaging technique that allows multiple-depth visualization as well as a 3D image with high resolution. (Fig. 4) illustrates LCSM photos of the brain uptake of Rhodamine solution and the dye-labeled PDA NPs. PDA NPs fluorescence was more intense when compared to the rhodamine solution demonstrating boosted dye uptake and penetration. Rhodamine-labeled PDA NPs proved successful penetration into the blood-brain barrier and brain tissue uptake. The rapid mucociliary clearance as well as the hydrophilicity of rhodamine dye solution might be responsible for the reduced permeation and hence brain uptake following I.N. administration^32^. The high penetration of PDA NPs is attributed to both PDA and sodium taurocholate. It was previously stated that PDA coating accelerated mucus penetration and enhanced cell uptake^33^. PDA polymer’s zwitterionic property with isoelectric point 4-4.5 allowed for potential penetration into both mucus and epithelial barriers. Also, PDA NPs minimized interactions with mucin owing to its negative charge and hence, facilitated mucus penetrability. Correspondingly, the negative charges allowed the interaction with the positive choline groups on the epithelium facilitating penetration into the epithelial barriers^34,35^. Sodium taurocholate has been identified as one of the most effective and safe penetration enhancers for intranasal formulation^36^.

### Effect of polydopamine nanoparticles on y maze

**Fig. 5:**
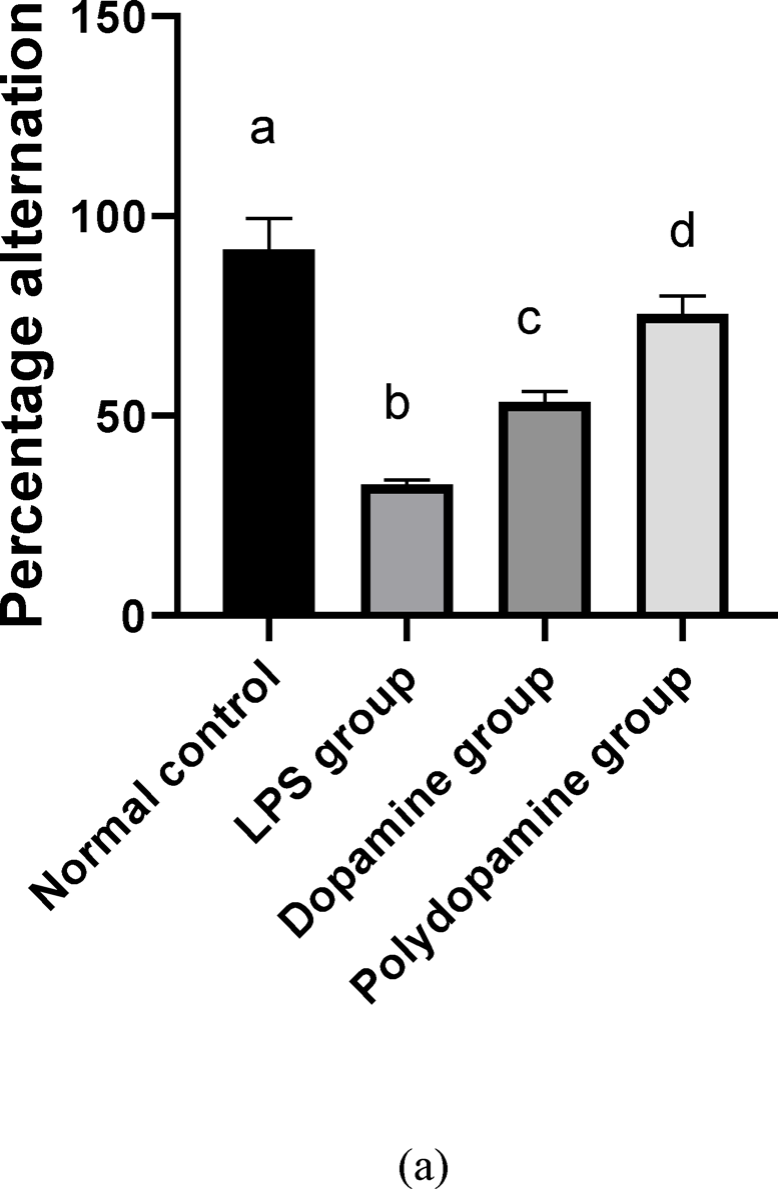

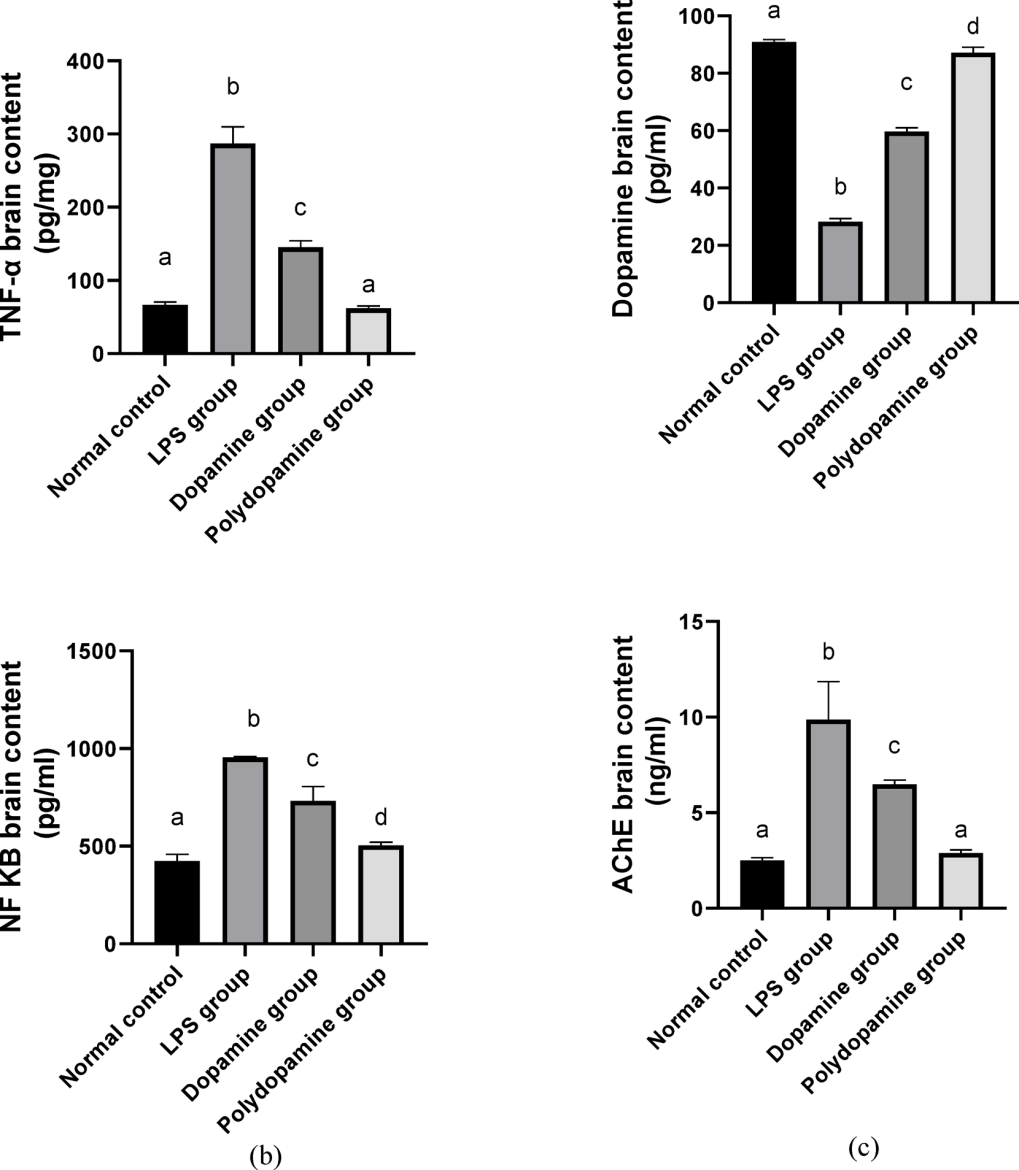
(**a**) Effect of polydopamine nanoparticles on percentage alteration in y-maze. The normal group received normal saline; the positive control group (LPS group) was induced by intraperitoneal lipopolysaccharide LPS (250 µg/kg) once daily for 1 week (IP); the dopamine group was treated simultaneously by LPS (250 µg/kg) and 100 ul of DA HCl solution (10 mg/ml) intranasal once daily for 7 days; the polydopamine group was treated simultaneously by LPS (250 µg/kg) and 100ul of PDA NPs (10 mg/ml) intranasal once a day for 7 days. The same letter means non-significant difference, while a different letter means significant difference at p < 0.05. (**b**) Effect of polydopamine nanoparticles on TNF and NF-κβ brain contents. The normal group received normal saline; the positive control group (LPS group) was induced by lipopolysaccharide LPS (250 µg/kg) once daily for 1 week intraperitoneal (IP); the dopamine group was treated simultaneously by LPS (250 µg/kg) and 100 ul of DA HCl solution (10 mg/ml) intranasal once daily for 7 days; the polydopamine group was treated simultaneously by LPS (250 µg/kg) and 100ul of PDA NPs (10 mg/ml) intranasal once a day for 7 days. The same letter means non-significant difference, while a different letter means significant difference at p < 0.05. (**c**) Effect of polydopamine nanoparticles on dopamine and AChE brain contents. The normal group received normal saline; the positive control group (LPS group) was induced by lipopolysaccharide LPS (250 µg/kg) once daily for 1 week intraperitoneal (IP); the dopamine group was treated simultaneously by LPS (250 µg/kg) and 100 ul of DA HCl solution (10 mg/ml) intranasal once daily for 7 days; the polydopamine group was treated simultaneously by LPS (250 µg/kg) and 100ul of PDA NPs (10 mg/ml) intranasal once a day for 7 days. The same letter means non-significant difference, while a different letter means significant difference at p < 0.05.

Lipopolysaccharide (LPS), a Gram-negative bacteria cell wall constituent, reduced activity and social contact, and food and drink consumption^37^. The Y- maze test is used to evaluate spatial working and recognition memories in rats. The current results revealed that LPS decreased Y-maze alteration by 64% inducing memory impairment compared to normal control, while the administration of DA HCl solution and the optimized PDA NPs produced a significant (*p* < 0.05) rise of Y-maze alteration by 63% and 130%, respectively, compared to LPS group (Fig. 5a). This memory deficiency of LPS has been allied to the accumulation of amyloid beta (Aβ)^38^. In addition, LPS produced neurobehavioral impairment by suppressing Nrf2/ARE/HO-1 antioxidant genes^39^. DA HCl and PDA NPs had previously proven their capabilities to counteract the reactive oxygen species, protect them from mitochondrial damage, and induce neurite outgrowth^40^. Consequently, a better neuroprotective effect was achieved by PDA NPs owing to their higher brain penetration.

### Effect of polydopamine nanoparticles on NF-κβ and TNF-α brain contents

Neuroinflammation was regarded as the chief pathogenic measure in AD^41^. Nuclear factor- κB (NF-κB) proinflammatory cytokine with amyloid beta peptides (Aβ) are highly expressed in AD patients’ brains. This overexpression of Aβ increased the acetylcholine (ACh) degradation that has a prime function in normal cognition and memory^42^. In addition, glial cells of CNS, are responsible for the neuron’s normal structure. During inflammation, glial cell upregulated and released inflammatory mediators inducing AD^43^. TNF-α is one of the inflammatory mediators that has a crucial role in the pathological progression of AD and participates in the dysfunction of the brain cells^44^. LPS acts as a modulator of neuroinflammation and imitates neurodegenerative disorders, including Parkinson’s disease, AD, and multiple sclerosis^45^. This study appraised the effect of PDA NPs on neuroinflammation induced by NF-κB and TNF-α (Fig. 5b). The results revealed the elevation in the expression of NF-κB and TNF-α after LPS injection in the brain by 125% and 331% respectively, compared to the normal control. However, administration of DA HCl solution and the optimized PDA NPs produced a significant (*p* < 0.05) reduction of NF-κB brain content by 23% and 47%, and TNF-α brain content by 49% and 78%, respectively, compared to LPS-treated positive control group. Moreover, treatment with PDA NPs restored TNF-α levels to their normal level.

### Effect of polydopamine nanoparticles on dopamine and acetylcholine esterase brain contents

DA is an important neurotransmitter in brain function and other neuroregulatory systems. However, its dysfunction participates to the memory impairment pathophysiology in AD^46^. Upon brain inflammation, microglia is activated accompanied by dopaminergic neuron loss^47^. LPS provokes oxidative stress, neuroinflammation, and loss of dopaminergic neurons^48^. However, the acetylcholine (ACh) neurotransmitter is involved in learning and memory actions. In AD patients, cognitive decline is linked with ACh deficiency^49^. Acetylcholinesterase (AChE) hydrolyzes acetylcholine into acetic acid and choline which is increased in AD patients^50^. In the current study, DA HCl solution and the optimized PDA NPs administration exhibited a reduction in AChE brain content by 34% and 71% and elevated the expression of dopamine by 112% and 209% respectively, compared to the LPS-treated group. Moreover, treatment with PDA NPs restored AChE levels to their normal level (Fig. 5c). These findings suggested that PDA NPs had neuroprotective activity by decreasing pro-inflammatory cytokine TNF-α, which in turn was responsible for the accumulation of Aβ plaque and neuronal loss. On the other hand, inhibiting the inflammation by PDA NPs caused a reduction in dopaminergic neuron degeneration.

### Histopathological results

**Fig. 6:**
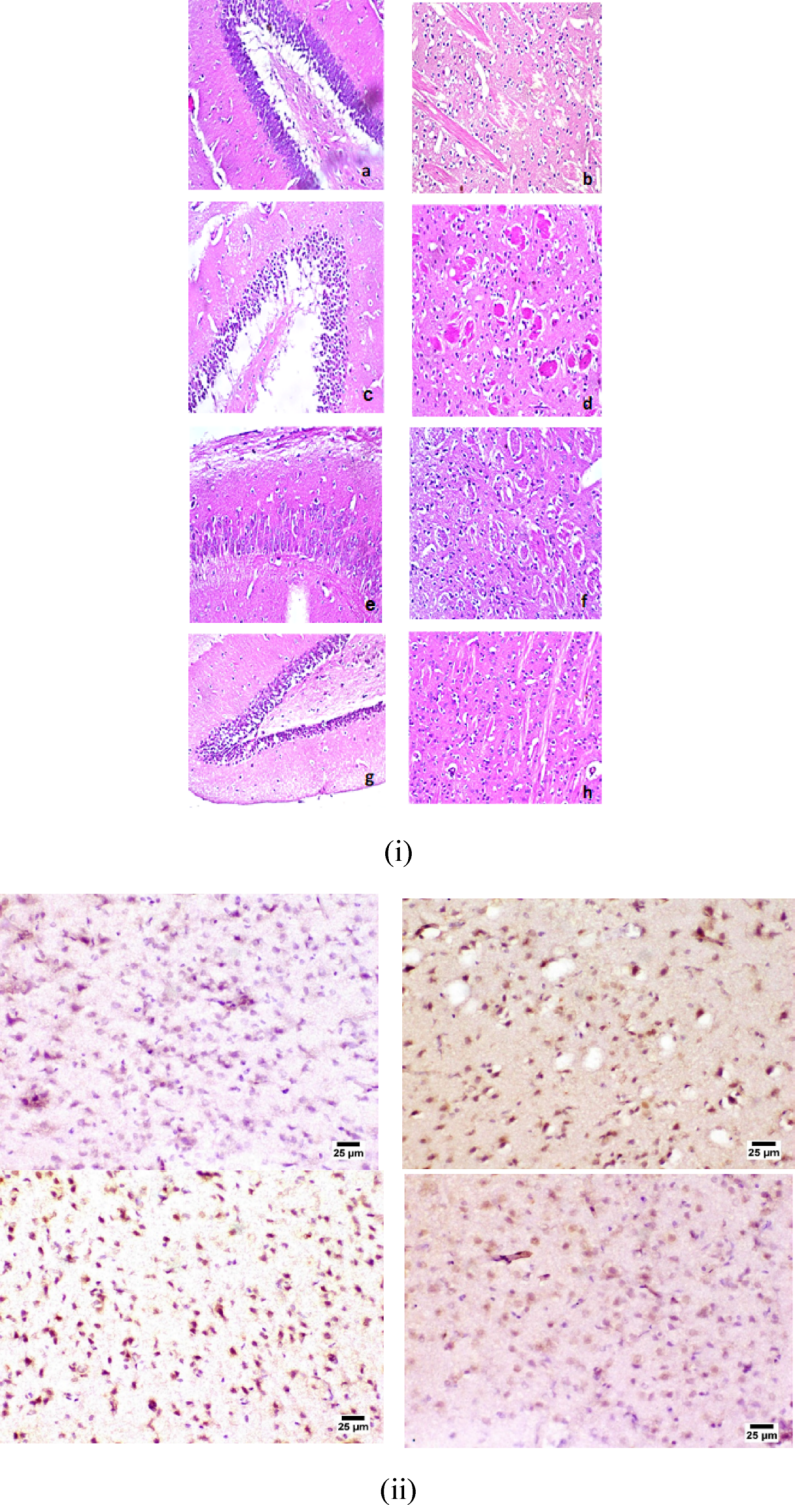
(**i**) Photomicrograph of the brain, (a, b) normal control group showing normal structure, (c, d) positive control group showing numerous degenerating neurons (e, f) DA- HCl solution treated group showing many degenerating neurons, (g, h) polydopamine nanoparticles treated group showing few degenerating neurons (H&E X40). (**ii**) Photomicrograph of brain, (a) Normal control group showing normal AChE expression, (b) Positive control group showing marked elevation in AChE expression, (c) Dopamine solution treated group showing an increase in AChE expression, and (d) Polydopamine nanoparticles treated group showing normal AChE expression (Immune staining).

The hippocampus and striatum of control group showed no histopathological alteration, and normal histological structure of the neurons, illustrated in (Fig. 6i(a and b)) respectively. However, the hippocampus of LPS group (positive control group) showed nuclear pyknosis and degeneration in most neurons and most of the neurons of striatum showed nuclear pyknosis and degeneration associated with multiple focal eosinophilic plaques formation in between (Fig. 6i(c-d)) respectively. In the DA HCl solution treated group, the hippocampus remained histologically unaltered (Fig. 6i(e). This may be due to the presence of dopamine receptors. Striatum showed focal eosinophilic plaques formation detected in between the neurons (Figure.6i(f)). The group treated with PDA NPs demonstrated a neuroprotective profile where; the hippocampus and striatum showed no histopathological alteration (Fig. 6i(g) and 6i(h)) respectively.

### Expression of acetylcholine esterase (AChE)

Our results revealed that the normal control group showed normal levels of AChE in the cerebral cortex. Meanwhile, marked elevation in its positive staining was detected in the positive control group. The group treated with dopamine solution exhibited a moderate increase in AChE expression compared to the positive control group. Finally, the group received PDA NPs treatment revealed normal levels of AChE positive staining (Fig.6ii).

## Conclusion

PDA NPs were synthesized via polymerization of DA HCl in combination with different types of surfactants. A mixed full factorial design (4^1^*2^2^) was employed for screening the amount of DA HCl, pH and surfactant type. The optimized formulation was composed of 10 mg dopamine, Tris- HCl buffer at pH = 10 using sodium taurocholate as a surfactant. The optimized formulation displayed a spherical appearance with a smooth surface. The MTT results reflected the biocompatibility and safety of PDA NPs on epithelial cell lines with IC_50_ = 397.4 µg/ mL. Enhanced permeation of PDA NPs was proved by brain tissue uptake study as visualized by CLSM. PDA NPs significantly decreased NF-κB, TNFα, and acetylcholine esterase (AChE) and showed an elevated dopamine expression and ameliorated neuron degeneration. Thus, PDA NPs could be considered as a potential neuroprotective candidate in management of AD.

## Supplementary Information

Below is the link to the electronic supplementary material.

Supplementary Material 1

## Data Availability

All generated or analyzed data throughout the study are furnished upon request by the corresponding author.
