# Supplementary material for: Evaluation of the neuroprotective potential of optimized intranasal polydopamine nanoparticles in a lipopolysaccharide-induced rat model for Alzheimer’s disease management
Source: Sci Rep. 2025 Nov 4;15:38491. doi: 10.1038/s41598-025-22844-z (PMC12586645; doi:10.1038/s41598-025-22844-z)

Fig. S1: FTIR spectra of pure dopamine hydrochloride (DA HCl), Sodium taurocholate, physical mixture (PM), and polydopamine nanoparticles (PDA NPs).


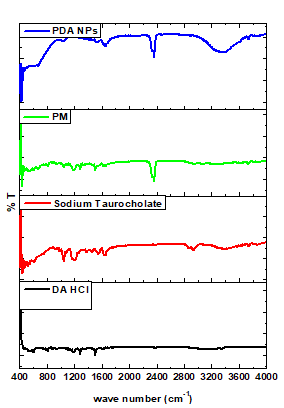

Supplement: Supplementary file 1 — Supplementary Material 1 [file 41598_2025_22844_MOESM1_ESM.docx]
